# The long-term outcomes in adolescent and young adult patients with colorectal cancer -A multicenter large-scale cohort study

**DOI:** 10.7150/jca.36721

**Published:** 2020-03-04

**Authors:** Yujiro Nakayama, Hiroshi Kobayashi, Hidetaka Kawamura, Rie Matsunaga, Yukitoshi Todate, Yoshinao Takano, Keiichi Takahashi, Shinichi Yamauchi, Kenichi Sugihara, Michitaka Honda

**Affiliations:** 1 Department of Minimally Invasive Surgical and Medical Oncology, Fukushima Medical University, Fukushima.; 2 Department of Surgery, Southern Tohoku General Hospital, Koriyama.; 3 Department of Surgery, Tokyo Metropolitan Cancer and Infectious Diseases Center Komagome Hospital, Tokyo.; 4 Department of Surgery, Tokyo Medical and Dental University, Tokyo.

**Keywords:** Colorectal Neoplasms, adolescent and young adult, prognosis, middle aged, adjuvant therapy

## Abstract

**Introduction**: The prognosis of adolescent and young adult (AYA) patients with colorectal cancer (CRC) is still unclear. The aim of this study was to investigate the clinical features and prognosis in AYA patients compared with middle- aged patients.

**Methods**: Participants were identified from a clinical database of the multicenter cohort in Japan. The AYA group was defined as those <40 years of age, whereas the middle-aged group was defined in 10-year ranges around the median age of all patients. The primary outcome was the overall survival (OS), and the secondary outcome was the recurrence-free survival (RFS).

**Results**: A total of 502 patients were enrolled as the AYA group, and 7222 patients between 65 and 74 years of age were identified as the middle-aged group. The OS of colon cancer in stages II and III was significantly better in the AYA group (p = 0.033, 0.006, respectively) than in the middle-aged groups. There were no significant differences in the OS of rectal cancer in stages II and III between the two groups.

**Conclusion**: The prognosis of AYA patients with CRC was the same or better than that in middle-aged patients.

## Introduction

Several previous reports have suggested that a younger age is an independent predictor of a poor prognosis among patients with colorectal cancer (CRC) 1-4. Generally speaking, few patients <50 years of age undergo periodic screening examinations, such as colonoscopy or fecal occult blood test, and are thus often diagnosed with advanced stage CRC with a severe symptomatic condition (e.g. obstructive colitis, anemia or bleeding) 5. In addition, regarding the clinicopathological characteristics of CRC in adolescent and young adult (AYA) patients, previous reports have shown that the incidence is particularly common in men and in the left-side colon, frequently with a histologically poorly differentiated type. These findings are unfavorable for the oncological prognosis 5-7. However, whether or not a young age is an independent risk factor for a poor prognosis remains controversial.

We focused on AYA patients with CRC in the present study to answer the clinical question of whether or not a standard therapeutic strategy could be applied in relatively young patients. Reflecting the rapid graying of society, the mean age of patients with CRC has been increasing in recent years. Although AYA patients might be disadvantaged to some degree oncologically, they retain some advantages with regard to treatment, as there are generally fewer comorbidities and patients have a higher tolerability for surgery or other aggressive multidisciplinary approaches than older patients. If younger patients truly have a poorer prognosis than older ones, they will require a more aggressive treatment strategy than the standard therapy according to clinical guidelines. The impact of a young age on the prognosis of CRC is therefore a relevant issue meriting investigation.

In the present study, we compared the survival outcomes of AYA patients with CRC with those of middle-age patients after adjusting for the TNM stage and other confounding factors. Our hypothesis was that the age was not an independent risk for the survival of patients with CRC, and we verified this theory using a multi-center large-scale database. Through this study, we hope to provide relevant information that can aid physicians in selecting suitable treatment strategies.

## Methods

### Participants and cohort development

We identified AYA and middle-aged patients from the database established by the Japanese Study Group for Postoperative Follow-up of Colorectal Cancer (JFUP-CRC), consisting of 23 hospitals that are mainly university hospitals and cancer-specialized hospitals throughout Japan. During the 12-year period from 1997 to 2008, consecutive patients who underwent radical resection for CRC were isolated from this database. The inclusion criteria of this study were as follows: patients who underwent radical surgery with lymph node dissection for CRC, had histologically proven adenocarcinoma and were diagnosed with pathological stage 0 to III disease. Patients who have missing data on their diagnostic age and follow-up findings were excluded from the analyses.

The AYA group was defined as those <40 years of age, which is a cut-off commonly used in studies related to AYA patients with cancer 8-10. Furthermore, the middle-aged group was defined as those with a median age of around 10 years. This study was approved by the institutional review boards or ethics committee at each hospital. The cancer staging was based on the seventh Union for International Cancer Control (UICC) TNM classification.

### Study design and data collection

This study is a multi-center, retrospective cohort study. We collected the demographic characteristics of the patients, histopathological characteristics of the tumors and data related to surgery or chemotherapy. Data on patient demographics included age, gender and serum levels of tumor markers, such as CEA and CA19-9. Tumor characteristics included TNM factor, location, size, histologic type, microscopic lymphatic duct and vessel invasion. Perioperative treatment information was also collected.

### Outcome and Statistical analyses

The primary outcome was the overall survival (OS), and the secondary outcomes were the recurrence-free survival (RFS), tumor characteristics, implementation of adjuvant chemotherapy, and patterns of recurrence. The survival time was evaluated by the stage-stratified analyses between the AYA and middle-aged group using the Kaplan-Meier method and Log-rank test. The hazard ratios (HRs) and 95% confidence intervals (CIs) were estimated using the Cox proportional hazards model as primary analyses. The descriptive statistics were evaluated in other secondary outcomes, and as necessary, continuous variables were compared using Student's *t*-test and categorical variables using Fisher's exact test. All statistical tests were two-sided, and P values of ≤0.05 were considered to indicate statistical significance. All analyses were performed with STATA version 14 (Texas, USA).

## Results

### Development of study cohort

During the study period, 21,242 patients underwent surgery for CRC in participating hospitals. The median age of all patients was 65 years old, so the “middle-aged group” was set as those 65 to 74 years of age in this study. After data extraction and cleaning according to the criteria, 502 and 7,222 patients were ultimately isolated for the AYA group and the middle-aged group, respectively. The median observation periods in the AYA and middle-aged groups were 76.7 (range 1-166) and 74.4 (1-220) months, respectively. The median observational period was 2221 days (73.0 months) in the AYA group and 2199 days (72.2 months) in the middle-aged group.

### Patients' characteristics

Table 1 shows the characteristics of the two groups. The median age in the AYA group is 35 (range 17-39) years. As patients with lymph node metastasis were more frequent in the AYA group, the proportion of stage III is significantly higher in the AYA group than in the middle-age group (42.8 vs. 36.2%, p<0.001).

Rectal cancer was more common in the AYA group than in the middle-aged group (54.4 vs. 37.5%, p<0.001). Among histological findings, undifferentiated tumors were significantly more common in the AYA group than in the middle-aged group (10.2% vs. 5.6%, p<0.001). No significant differences in the mean serum level of CEA or CA19-9 were noted between the two groups.

### Survival Outcomes

**Figure 1:**
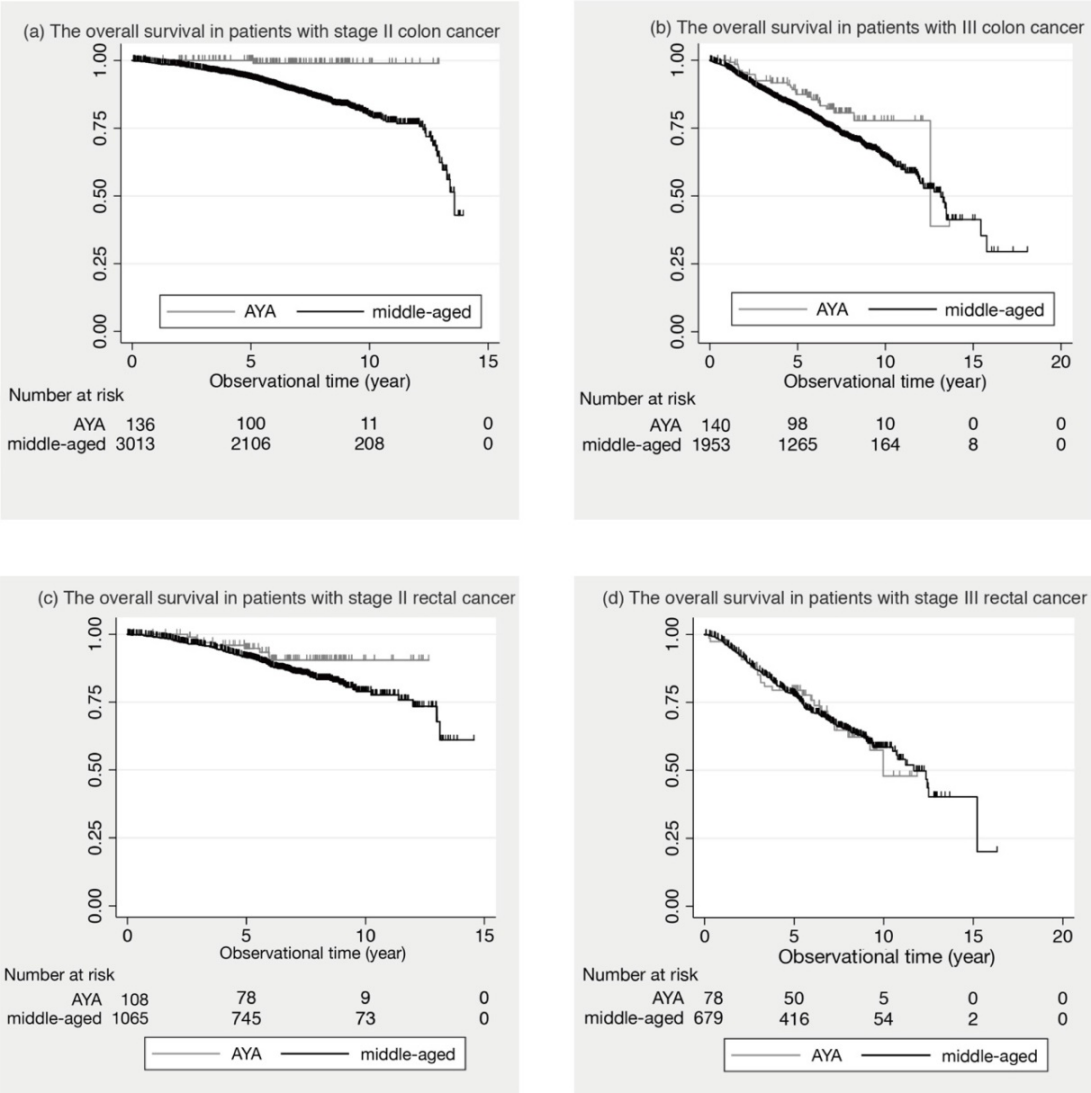
** The overall survival in stage II and III patients. (a)** The overall survival in patients with stage II colon cancer is shown. **(b)** The overall survival in patients with III colon cancer is shown. **(c)** The overall survival in patients with stage II rectal cancer is shown. **(d)** The overall survival in patients with stage III rectal cancer is shown.

Figure 1 shows the comparison of the survival curves between the AYA and middle-aged groups in colon and rectal cancer.

In stage II colon cancer, the 5-year OS was 100.0% in AYA patients and 93.6% (95% Confidential Intervals: 92.6 to 94.5) in middle-aged patients. In stage III colon cancer, 87.4% (95% C.I.: 80.3 to 92.1) in AYA and 82.2% (95% C.I.: 80.4 to 83.9) in middle-aged. In stage II and III colon cancer, the 5-year OS of the AYA group was significantly better than that of the middle-aged group (p= 0.033, p= 0.006, respectively). In contrast, in stage II and III rectal cancer, there was no significant difference in the 5-year OS between the two groups. (p= 0.109, p= 0.878, respectively).

In stage II rectal cancer, the 5-year RFS was significantly better than that of the middle-aged group (48.8%, 60.2%, respectively, p= 0.019). There were no significant differences in the 5-year RFS in stage II and III colon cancer, and stage III rectal cancer between the 2 groups (shown in supplemental figure).

### Adjuvant therapy

Table 2 shows the number of patients who underwent adjuvant therapy in stages II and III. Among both colon and rectal cancer patients with stage III disease, the proportions receiving adjuvant therapy were higher in the AYA group than in the middle-aged group.

### Recurrence patterns

Table 3 shows the patterns of recurrence during the follow-up period. There were no significant differences between the groups.

### Risk factors for overall survival

Table 4 showed the risk factors for overall survival. Hazard ratios of AYA were 1.9 (Confidential intervals: 1.29- 2.80) and 1.47 (Confidential intervals: 0.99- 2.18) in colon and rectal cancer patients, respectively.

## Discussion

In the present study, we demonstrated that a young age is not an independent risk for the long-term outcomes in CRC patients. In terms of oncological outcomes stratified pathological stage, the AYA group had a better OS of colon cancer than the middle-aged group, while the survival results for rectal cancer were equivalent between the two groups. In addition, we found that several clinical features—namely the incidence of rectal cancer, histologically poorly differentiated type and lymph node metastasis—were more frequent in AYA patients than in middle-aged patients.

Previous reports have suggested that a screening effect in middle-aged patients confers upon them a better prognosis than that of younger patients, as CRC tends to be found at an earlier stage in middle-aged patients than in younger ones 5-7. We therefore adjusted for the TNM stage and tumor location to ensure a proper comparison between the two groups. As a result, our findings implied that the AYA group had the same or a better prognosis than the older patients.

Based on the present results, we considered that a young age was not an independent risk factor for the long-term prognosis in CRC patients. However, we must bear in mind two important points when treating AYA patients: the increased incidence of lymph node metastasis and the greater proportion receiving adjuvant chemotherapy in the AYA group than in the middle-aged group. As poorly differentiated adenocarcinoma is more common in AYA patients than in older ones, the proportion with positive lymph nodes was higher in this group than in the middle-aged group. A similar situation has also been reported in young patients with gastric cancer 11. Thus, during radical resection, more rigorous lymph node dissection might be needed for young population.

In addition, possible recurrence should be considered during the postoperative follow-up. However, as young patients tend to have a relatively low incidence of comorbidities or postoperative complications, they are able to tolerate adjuvant therapy well. Indeed, a higher proportion of AYA patients than middle-aged patients received adjuvant therapy in our study (57.6% vs. 42.4%). Consequently, the prognosis of colon cancer was superior in the AYA group to that in the middle-aged group. However, of note: the OS and RFS of rectal cancer were almost the same in both groups despite more AYA patients receiving adjuvant chemotherapy than middle-aged patients. To improve the oncological outcome in AYA patients with rectal cancer, we should perform R0 resection with sufficient lymphadenectomy and increase the proportion of adjuvant treatment according to the guideline 12,13. In addition, a more aggressive regimen and concurrent radiotherapy might be required for high-risk patients. To answer our clinical question as to whether or not a standard therapeutic strategy could be applied for relatively young patients, adjuvant chemotherapy should be performed rigorously for stage III AYA patients, and a more aggressive regimen such as oxaliplatin-based chemotherapy might be considered for high risk stage II AYA patients 14.

A major limitation associated with the present study warrants mention. The lack of any data on a history of familial cancer, such as Lynch syndrome, which is known to be a good prognosis factor, is a weakness of this study. However, Myers et al. reported that CRC was common in young patients with no family history, as only 12% of patients in their cohort had a first-degree relative with CRC; this therefore likely had a limited influence on our results 5. Despite this limitation, our study had a larger sample size than previous reports, so we were able to answer our clinical question. Additionally, as the observational period was 10 years previously, the impact of new strategies for CRC, such as oxaliplatin-based adjuvant chemotherapy, molecular targeting therapy, radical hepatectomy and lung resection were not reflected in this study.

## Conclusions

We conclude that a young age is not an independent risk factor for the prognosis in patients with CRC.

## Supplementary Material

Supplementary figure.

## Figures and Tables

**Table 1:** Patients' characteristics

Characteristic		AYA n= 502	Percentage (%)	Middle aged (n= 7222)	Percentage (%)	*p* value
Age	median	35	-	69	-	-
Gender	male	285	(56.8)	4441	(61.5)	0.056
	female	217	(43.2)	2811	(38.9)	
Primary site	Right colon*	110	(21.9)	2110	(29.2)	<0.001
	Left colon**	119	(23.7)	2404	(33.3)	
	Rectum	273	(54.4)	2708	(37.5)	
pT	0/1	98	(19.5)	1226	(17.0)	0.052
	2	89	(17.7)	1188	(16.5)	
	3	235	(46.8)	3649	(50.5)	
	4a	53	(10.6)	904	(12.5)	
	4b	27	(5.4)	255	(3.5)	
pN	0	287	(57.2)	4610	(63.8)	<0.001
	1	136	(27.1)	1880	(26.0)	
	2	72	(14.3)	633	(8.8)	
	3	7	(1.4)	99	(1.4)	
Stage (UICC)	0	2	(0.4)	10	(0.1)	<0.001
	I	141	(28.1)	1969	(27.3)	
	IIA	106	(21.1)	2018	(27.9)	
	IIB	21	(4.2)	354	(4.9)	
	IIC	3	(0.6)	117	(1.6)	
	IIIA	81	(16.1)	351	(4.9)	
	IIIB	136	(27.1)	2302	(31.9)	
	IIIC	12	(2.4)	101	(1.4)	
Tumor size	mean (mm)	40	-	40	-	0.850
Histology	well/moderately	450	(89.6)	6811	(94.3)	<0.001
	mucinous/ poorly	51	(10.2)	402	(5.6)	
	unknown	1	(0.2)	9	(0.1)	
ly	negative	199	(39.6)	2834	(39.2)	0.887
	positive	298	(59.4)	4313	(57.8)	
	unknown	5	(1.0)	75	(1.0)	
v	negative	236	(47.0)	2968	(41.1)	0.002
	positive	258	(59.7)	4172	(57.8)	
	unknown	8	(1.6)	82	(1.1)	
Tumor maker	CEA (ng/dl)	3.0	-	2.9	-	0.265
	CA19-9 (ng/dl)	11.0	-	11.0	-	0.675

* including radiotherapy, Right colon includes cecal, ascending and transverse colon. Left colon includes discending and sigmoid colon. AYA; adolescents and young adults. UICC; Union for International Cancer Control.

**Table 2:** The proportion of patients who underwent adjuvant therapy

		AYA (n= 359)	Percentage (%)	Middle aged (n= 5243)	Percentage (%)	p value
						< 0.001
All stage II/III patients	Adjuvant	174	(48.5)	1796	(34.3)	
	No adjuvant	128	(35.7)	2441	(46.6)	
	Unknown	57	(15.9)	1006	(19.2)	
Colon	stage II					0.123
	Adjuvant	16	(27.1)	282	(17.1)	
	No adjuvant	36	(61.0)	1030	(62.4)	
	Unknown	7	(11.9)	338	(20.5)	
	stage III					0.003
	Adjuvant	73	(65.2)	827*	(49.7)	
	No adjuvant	23	(20.5)	530	(31.9)	
	Unknown	16	(14.3)	306	(18.4)	
Rectum	stage II					0.871
	Adjuvant	14	(19.7)	167*	(19.9)	
	No adjuvant	41	(57.7)	517	(61.6)	
	Unknown	16	(22.5)	155	(18.5)	
	stage III					0.013
	Adjuvant	71*	(60.7)	520*	(47.7)	
	No adjuvant	28	(23.9)	364	(33.4)	
	Unknown	18	(15.4)	207	(19.0)	

* including radiotherapy, Right colon includes cecal, ascending and transverse colon. Left colon includes discending and sigmoid colon. AYA; adolescents and young adults. UICC; Union for International Cancer Control.

**Table 3:** Recurrence patterns

Tumor location	Recurrence patterns	AYA (n= 502)	%	Middle aged (n= 7222)	%	p value
Colon	Liver	17	(48.6)	270	(43.1)	0.829
	Lung	7	(20.0)	122	(19.5)	
	Lymph node	2	(5.7)	76	(12.1)	
	Dissemination	3	(8.6)	82	(13.1)	
	Local	2	(5.7)	38	(6.1)	
	Others	2	(5.7)	26	(4.2)	
	Unknown	2	(5.7)	12	(1.9)	
Rectum	Liver	19	(29.7)	197	(33.1)	0.085
	Lung	17	(26.6)	167	(28.0)	
	Lymph node	3	(4.7)	63	(10.6)	
	Dissemination	3	(4.7)	13	(2.2)	
	Local	14	(21.9)	124	(20.8)	
	Others	6	(9.4)	19	(3.2)	
	Unknown	2	(3.1)	13	(2.2)	

AYA; adolescents and young adults.

**Table 4:** Risk factors for overall survival

	Variant	Hazard Ratio	95% Confidential Interval	*p* value
Colon	AYA	1.9	1.29- 2.80	0.001
	adjvant therapy	0.66	0.56- 0.78	< 0.001
	gender	0.59	0.51- 0.68	< 0.001
	histology	1.37	1.07- 1.75	0.012
	pT	1.35	1.25- 1.45	< 0.001
	pN	1.82	1.65-1.99	< 0.001
Rectum	AYA	1.47	0.99- 2.18	0.058
	adjvant therapy	0.89	0.71- 1.12	0.324
	gender	0.76	0.6- 0.96	0.022
	histology	2.04	1.48- 2.83	< 0.001
	pT	1.67	1.49- 1.87	< 0.001
	pN	1.46	1.28- 1.68	< 0.001

AYA; Adolescents and young adults.

## Abbreviations

AYA: adolescent and young adult
CIs: confidence intervals
CRC: colorectal cancer
HRs: hazard ratios
OS: overall survival
RFS: recurrence-free survival
UICC: Union for International Cancer Control
