# Supplementary material for: The long-term outcomes in adolescent and young adult patients with colorectal cancer -A multicenter large-scale cohort study
Source: J Cancer. 2020 Mar 4;11(11):3180–5. doi: 10.7150/jca.36721 (PMC7097932; doi:10.7150/jca.36721)
Supplement: Supplementary file 1 — Supplementary figure. [file jcav11p3180s1.pdf]

Supplemental figure

Figure legend

Supplement figure

The recurrence-free survival in stage II and III patients. (a) The recurrence-free survival in patients with stage II colon cancer is shown. (b) The recurrence-free survival in patients with III colon cancer is shown. (c) The recurrence-free survival in patients with stage II rectal cancer is shown. (d) The recurrence-free survival in patients with stage III rectal cancer is shown.

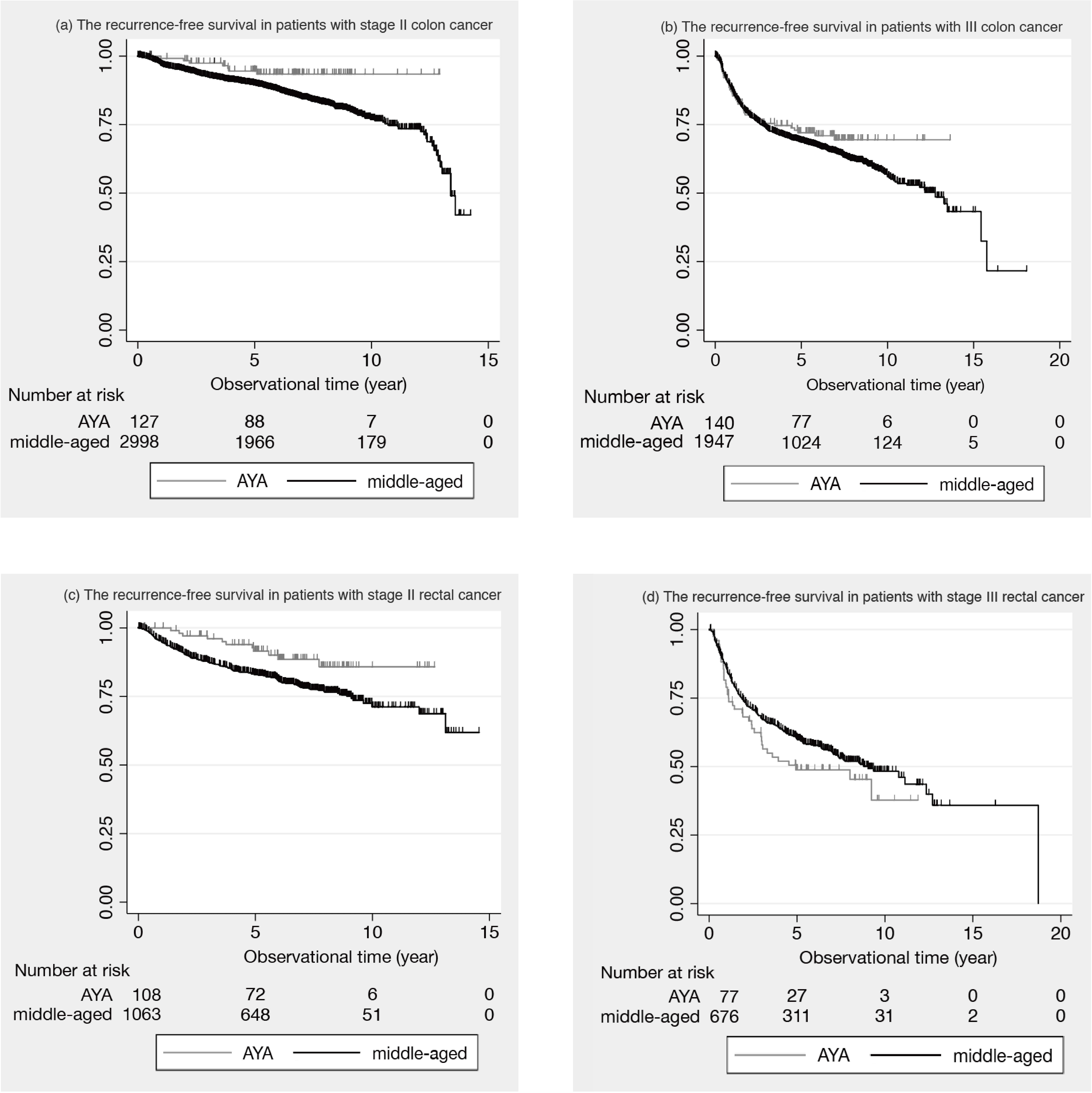
(a) The recurrence-free survival in patients with stage II colon cancer
1.00
0.75
0.50
0.25
0.00
0
5
10
15
Observational time (year)
Number at risk
AYA 127 88 7 0
middle-aged 2998 1966 179 0
AYA
middle-aged
(b) The recurrence-free survival in patients with III colon cancer
1.00
0.75
0.50
0.25
0.00
0
5
10
15
20
Observational time (year)
Number at risk
AYA 140 77 6 0 0
middle-aged 1947 1024 124 5 0
AYA
middle-aged
(c) The recurrence-free survival in patients with stage II rectal cancer
1.00
0.75
0.50
0.25
0.00
0
5
10
15
Observational time (year)
Number at risk
AYA 108 72 6 0
middle-aged 1063 648 51 0
AYA
middle-aged
(d) The recurrence-free survival in patients with stage III rectal cancer
1.00
0.75
0.50
0.25
0.00
0
5
10
15
20
Observational time (year)
Number at risk
AYA 77 27 3 0 0
middle-aged 676 311 31 2 0
AYA
middle-aged
